# (7*R*,8*S*,9*S*,12*S*)-1-Benz­yloxy-13,14-didehydro-12-hy­droxy-2,13-dimeth­oxy-*N*-methyl­morphinane

**DOI:** 10.1107/S1600536811037226

**Published:** 2011-09-17

**Authors:** Xing-Liang Zheng, Ning-Fei Jiang, Dan Luo, Hong-Sheng Gao, Ai-Shun Ding

**Affiliations:** a School of Chemistry and Biological Engineering, Changsha University of Science & Technology, Changsha 410114, People’s Republic of China

## Abstract

In the title compound, C_26_H_31_NO_4_, a sinomenine derivative, the angle between the two aromatic rings is 53.34 (4)°. The N-containing ring is in a chair conformation, while the other two non-planar rings are in a half-boat conformation. In the crystal, mol­ecules are linked by O—H⋯N inter­actions into a *C*(8) chain along [100].

## Related literature

For background to the biological effects (such as anti-inflammatory, analgesic, anti-rheumatoid arthritis and arrhythmia, lowering of blood pressure and immune function) of sinomenine derivatives and other related compounds, see: Liu *et al.* (1994 ▶, 1996 ▶, 1997 ▶); Mark *et al.* (2003 ▶); Ye *et al.* (2004 ▶). For related structures, see: Li *et al.* (2009 ▶); Batterham *et al.* (1965 ▶); Zheng & Jiang (2010 ▶); Zheng *et al.* (2011 ▶). For hydrogen-bond motifs, see: Bernstein *et al.* (1995 ▶). For puckering parameters, see: Cremer & Pople (1975 ▶). For the synthesis of 9*S*,13*R*,14*S*)-7,8-didehydro-4-benz­yloxy-3,7-dimeth­oxy-17-methyl­morphinan-6-one, a starting material in the preparation of the title compound, see: Hitotsuyanagi *et al.* (1995 ▶).
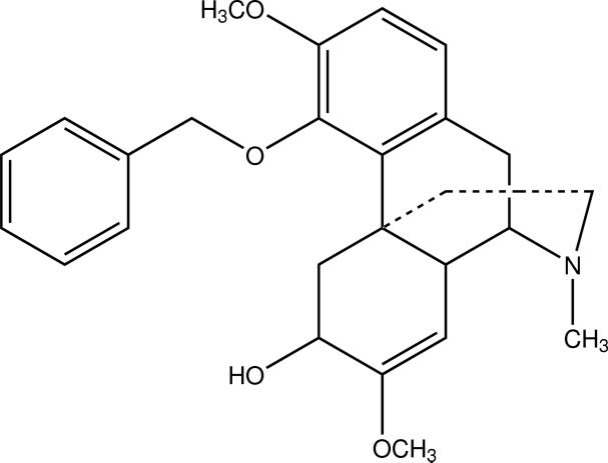


## Experimental

### 

#### Crystal data

C_26_H_31_NO_4_
*M* *_r_* = 421.52
Triclinic, 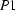

*a* = 7.7191 (2) Å
*b* = 8.5100 (2) Å
*c* = 9.9630 (2) Å
α = 79.971 (1)°
β = 67.663 (1)°
γ = 64.605 (1)°
*V* = 546.81 (2) Å^3^
*Z* = 1
Cu *K*α radiation
μ = 0.69 mm^−1^
*T* = 133 K
0.22 × 0.18 × 0.16 mm

#### Data collection

Bruker APEXII CCD diffractometer
Absorption correction: multi-scan (*SADABS*; Bruker, 2000 ▶) *T*_min_ = 0.864, *T*_max_ = 0.898
9789 measured reflections
2925 independent reflections
2918 reflections with *I* > 2σ(*I*)
*R*_int_ = 0.020

#### Refinement

*R*[*F* ^2^ > 2σ(*F* ^2^)] = 0.028
*wR*(*F* ^2^) = 0.075
*S* = 1.07
2925 reflections
320 parameters
3 restraints
H atoms treated by a mixture of independent and constrained refinement
Δρ_max_ = 0.16 e Å^−3^
Δρ_min_ = −0.21 e Å^−3^
Absolute structure: Flack (1983 ▶), 1075 Friedel pairs
Flack parameter: −0.09 (14)

### 

Data collection: *APEX2* (Bruker, 2000 ▶); cell refinement: *SAINT* (Bruker, 2000 ▶); data reduction: *SAINT*; program(s) used to solve structure: *SHELXS97* (Sheldrick, 2008 ▶); program(s) used to refine structure: *SHELXL97* (Sheldrick, 2008 ▶); molecular graphics: *SHELXTL* (Sheldrick, 2008 ▶); software used to prepare material for publication: *SHELXTL*.

## Supplementary Material

Crystal structure: contains datablock(s) global, I. DOI: 10.1107/S1600536811037226/bx2370sup1.cif

Structure factors: contains datablock(s) I. DOI: 10.1107/S1600536811037226/bx2370Isup2.hkl

Additional supplementary materials:  crystallographic information; 3D view; checkCIF report

## Figures and Tables

**Table 1:** Hydrogen-bond geometry (Å, °)

*D*—H⋯*A*	*D*—H	H⋯*A*	*D*⋯*A*	*D*—H⋯*A*
O3—H3⋯N1^i^	0.81 (3)	2.20 (3)	2.8966 (17)	145 (2)

Symmetry code: (i) 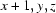.

**Fig. 1.**
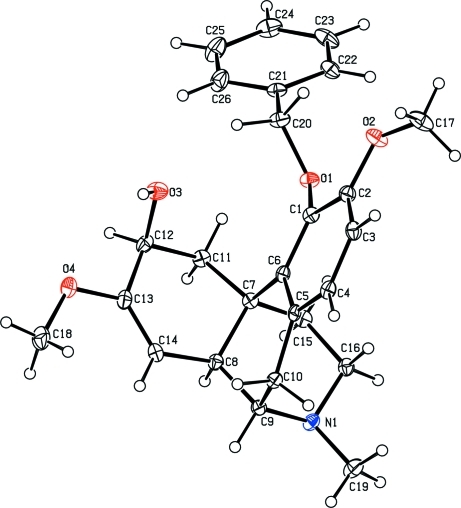
The molecular structure of the title compound showing 30% probability displacement ellipsoids.

**Fig. 2.**
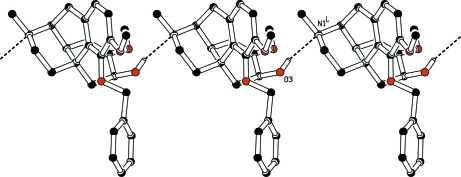
Part of the crystal structure showing the formation of a C(8) chain along [100]. Hydrogen bond shown as dashed lines. The hydrogen atom no involved to hydrogen bond were omitted by clarity. Symmetry code: (i) x + 1, y, z.

## supplementary crystallographic information

### Comment

We have synthesized a new sinomenine derivative, the title compound (7*R*,8*S*,9*S*,12*S*)-13,14-didehydro-1-benzyloxy-*N*-methyl-2,13- dimethoxy-12-hydroxymorphinane, C_26_H_31_NO_4_ and report its crystal structure. The molecular structure of the title compound is shown in Fig. 1. The angle between the two aromatic planar rings is 53.34 (4)°. The N-containing ring approximates the chair conformation (Q_T_ = 0.6052 (17) Å θ = 171.70 (16)° & φ = 330.4 (11)°) while other non-planar rings; C5—C10 & C7/C8/C11—C14 approximate have half-boat conformation (Q_T_ = 0.5304 (17) Å θ =48.94 (18)° & φ = 207.8 (2)°; Q_T_ = 0.5065 (18) Å, θ = 51.5 (2)° & φ = 347.7 (3)° respectively, Cremer & Pople, 1975). In the crystal structure de molecules are linked by O—H···N interactions into a chain along [100] with set-graph notation C(8), (Bernstein *et al.*, 1995), Fig. 2, Table 1. Similar features have been described in related compounds (Zheng & Jiang, 2010; Zheng *et al.*, 2011; Li *et al.*, 2009; Batterham *et al.*, 1965).

### Experimental

The title compound was obtained by reducing (9*S*,13*R*,14*S*)-7,8-Didehydro-4-benzyloxy-3,7-dimethoxy-17-methyl-morphinan-6-one (which was synthesized by Hitotsuyanagi *et al.*, 1995) with lithium aluminium tetrahydride. Colorless blocks were grown from a ethyl acetate–hexane solution.

### Refinement

H atoms were positioned geometrically, with C—H = 0.95 (aromatic CH), 0.98 (methyl CH_3_), 0.99 (methylene CH_2_) or 1.00 Å (methine CH), and were constrained to ride on their parent atoms, with *U*_iso_(H) = 1.2*U*_eq_(carrier C) or *U*_iso_(H) = 1.5*U*_eq_ (carrier C17 C18 C19). H atom attached to O atom was refined isotropically. 1141 Friedel pairs were used for the Flack parameter refinement.

### Crystal data

**Table:**

C_26_H_31_NO_4_	*Z* = 1
*M**_r_* = 421.52	*F*(000) = 226
Triclinic, *P*1	*D*_x_ = 1.280 Mg m^−^^3^
Hall symbol: P 1	Cu *K*α radiation, λ = 1.54178 Å
*a* = 7.7191 (2) Å	Cell parameters from 9563 reflections
*b* = 8.5100 (2) Å	θ = 4.8–67.7°
*c* = 9.9630 (2) Å	µ = 0.69 mm^−^^1^
α = 79.971 (1)°	*T* = 133 K
β = 67.663 (1)°	Block, colourless
γ = 64.605 (1)°	0.22 × 0.18 × 0.16 mm
*V* = 546.81 (2) Å^3^	

### Data collection

**Table:**

Bruker APEXII CCD diffractometer	2925 independent reflections
Radiation source: fine-focus sealed tube	2918 reflections with *I* > 2σ(*I*)
graphite	*R*_int_ = 0.020
φ and ω scans	θ_max_ = 65.0°, θ_min_ = 4.8°
Absorption correction: multi-scan (*SADABS*; Bruker, 2000)	*h* = −9→8
*T*_min_ = 0.864, *T*_max_ = 0.898	*k* = −9→10
9789 measured reflections	*l* = −11→11

### Refinement

**Table:**

Refinement on *F*^2^	Secondary atom site location: difference Fourier map
Least-squares matrix: full	Hydrogen site location: inferred from neighbouring sites
*R*[*F*^2^ > 2σ(*F*^2^)] = 0.028	H atoms treated by a mixture of independent and constrained refinement
*wR*(*F*^2^) = 0.075	*w* = 1/[σ^2^(*F*_o_^2^) + (0.0495*P*)^2^ + 0.074*P*] where *P* = (*F*_o_^2^ + 2*F*_c_^2^)/3
*S* = 1.07	(Δ/σ)_max_ < 0.001
2925 reflections	Δρ_max_ = 0.16 e Å^−^^3^
320 parameters	Δρ_min_ = −0.21 e Å^−^^3^
3 restraints	Absolute structure: Flack (1983), 1075 Friedel pairs
Primary atom site location: structure-invariant direct methods	Flack parameter: −0.09 (14)

### Special details

**Table:**

Geometry. All e.s.d.'s (except the e.s.d. in the dihedral angle between two l.s. planes) are estimated using the full covariance matrix. The cell e.s.d.'s are taken into account individually in the estimation of e.s.d.'s in distances, angles and torsion angles; correlations between e.s.d.'s in cell parameters are only used when they are defined by crystal symmetry. An approximate (isotropic) treatment of cell e.s.d.'s is used for estimating e.s.d.'s involving l.s. planes.
Refinement. Refinement of *F*^2^ against ALL reflections. The weighted *R*-factor *wR* and goodness of fit *S* are based on *F*^2^, conventional *R*-factors *R* are based on *F*, with *F* set to zero for negative *F*^2^. The threshold expression of *F*^2^ > σ(*F*^2^) is used only for calculating *R*-factors(gt) *etc*. and is not relevant to the choice of reflections for refinement. *R*-factors based on *F*^2^ are statistically about twice as large as those based on *F*, and *R*- factors based on ALL data will be even larger.

### Fractional atomic coordinates and isotropic or equivalent isotropic displacement parameters (Å^2^)

**Table:**

	*x*	*y*	*z*	*U*_iso_*/*U*_eq_	
N1	−0.1846 (2)	0.69043 (16)	0.42381 (14)	0.0213 (3)	
O1	0.20888 (17)	0.54646 (13)	0.78285 (11)	0.0221 (3)	
O2	0.1468 (2)	0.84505 (15)	0.88590 (12)	0.0311 (3)	
O3	0.6444 (2)	0.43153 (16)	0.48351 (13)	0.0299 (3)	
H3	0.699 (5)	0.493 (4)	0.431 (3)	0.048 (7)*	
O4	0.7533 (2)	0.42073 (17)	0.17433 (13)	0.0325 (3)	
C1	0.1806 (2)	0.6935 (2)	0.69393 (16)	0.0196 (3)	
C2	0.1435 (3)	0.8511 (2)	0.74825 (17)	0.0224 (3)	
C3	0.0987 (3)	1.0021 (2)	0.66552 (17)	0.0227 (3)	
H3A	0.0850	1.1071	0.6974	0.027*	
C4	0.0742 (2)	0.99827 (19)	0.53580 (16)	0.0206 (3)	
H4	0.0378	1.1031	0.4812	0.025*	
C5	0.1011 (2)	0.84563 (19)	0.48291 (15)	0.0178 (3)	
C6	0.1651 (2)	0.68794 (19)	0.55900 (16)	0.0180 (3)	
C7	0.1781 (2)	0.52104 (19)	0.50805 (15)	0.0186 (3)	
C8	0.1850 (2)	0.53675 (19)	0.34957 (15)	0.0191 (3)	
H8	0.1607	0.4365	0.3328	0.023*	
C9	0.0109 (2)	0.70153 (19)	0.32873 (16)	0.0194 (3)	
H9	0.0182	0.7046	0.2260	0.023*	
C10	0.0495 (2)	0.85695 (19)	0.34874 (16)	0.0197 (3)	
H10A	0.1631	0.8670	0.2625	0.024*	
H10B	−0.0738	0.9644	0.3531	0.024*	
C11	0.3642 (3)	0.35419 (18)	0.51321 (16)	0.0215 (3)	
H11A	0.3646	0.3349	0.6140	0.026*	
H11B	0.3489	0.2546	0.4878	0.026*	
C12	0.5705 (3)	0.3557 (2)	0.41207 (18)	0.0241 (3)	
H12	0.6691	0.2319	0.3919	0.029*	
C13	0.5602 (3)	0.4429 (2)	0.26876 (17)	0.0238 (3)	
C14	0.3887 (3)	0.5234 (2)	0.23985 (15)	0.0225 (3)	
H14	0.3944	0.5747	0.1464	0.027*	
C15	−0.0260 (3)	0.50452 (19)	0.60092 (16)	0.0210 (3)	
H15A	−0.0259	0.3980	0.5737	0.025*	
H15B	−0.0401	0.4940	0.7047	0.025*	
C16	−0.2061 (2)	0.6625 (2)	0.57832 (16)	0.0212 (3)	
H16A	−0.3340	0.6454	0.6335	0.025*	
H16B	−0.2155	0.7670	0.6162	0.025*	
C17	0.0786 (4)	1.0089 (3)	0.9494 (2)	0.0391 (5)	
H17A	0.172 (4)	1.067 (3)	0.899 (3)	0.042 (6)*	
H17B	−0.069 (4)	1.089 (3)	0.950 (2)	0.032 (5)*	
H17C	0.082 (4)	0.984 (3)	1.044 (3)	0.043 (6)*	
C18	0.7681 (3)	0.4831 (3)	0.0298 (2)	0.0387 (5)	
H18A	0.903 (5)	0.467 (3)	−0.018 (3)	0.051 (7)*	
H18B	0.692 (4)	0.609 (3)	0.029 (2)	0.042 (6)*	
H18C	0.725 (4)	0.419 (3)	−0.020 (3)	0.044 (6)*	
C19	−0.3610 (3)	0.8391 (2)	0.4052 (2)	0.0307 (4)	
H19A	−0.393 (4)	0.944 (3)	0.456 (2)	0.042 (6)*	
H19B	−0.477 (4)	0.811 (3)	0.452 (2)	0.037 (6)*	
H19C	−0.340 (4)	0.871 (3)	0.304 (3)	0.038 (6)*	
C20	0.3930 (3)	0.4804 (2)	0.81955 (18)	0.0264 (4)	
H20A	0.3872	0.5670	0.8776	0.032*	
H20B	0.5140	0.4588	0.7299	0.032*	
C21	0.4100 (3)	0.3140 (2)	0.90526 (17)	0.0231 (3)	
C22	0.2758 (3)	0.3134 (2)	1.04523 (17)	0.0263 (4)	
H22	0.1650	0.4186	1.0858	0.032*	
C23	0.3022 (3)	0.1601 (2)	1.12647 (17)	0.0335 (4)	
H23	0.2098	0.1610	1.2224	0.040*	
C24	0.4635 (3)	0.0053 (2)	1.0678 (2)	0.0349 (4)	
H24	0.4828	−0.0992	1.1240	0.042*	
C25	0.5956 (3)	0.0046 (3)	0.9271 (2)	0.0405 (5)	
H25	0.7050	−0.1009	0.8859	0.049*	
C26	0.5683 (3)	0.1574 (3)	0.8465 (2)	0.0347 (4)	
H26	0.6587	0.1557	0.7497	0.042*	

### Atomic displacement parameters (Å^2^)

**Table:**

	*U*^11^	*U*^22^	*U*^33^	*U*^12^	*U*^13^	*U*^23^
N1	0.0167 (8)	0.0222 (7)	0.0249 (6)	−0.0089 (6)	−0.0070 (5)	0.0024 (5)
O1	0.0253 (7)	0.0243 (5)	0.0217 (5)	−0.0144 (5)	−0.0113 (5)	0.0073 (4)
O2	0.0463 (9)	0.0300 (6)	0.0225 (6)	−0.0164 (6)	−0.0159 (5)	−0.0007 (5)
O3	0.0305 (7)	0.0379 (7)	0.0307 (6)	−0.0217 (6)	−0.0133 (5)	0.0053 (5)
O4	0.0190 (7)	0.0432 (7)	0.0286 (6)	−0.0103 (6)	−0.0016 (5)	−0.0056 (5)
C1	0.0192 (10)	0.0199 (7)	0.0201 (7)	−0.0102 (7)	−0.0065 (6)	0.0042 (6)
C2	0.0209 (10)	0.0267 (8)	0.0205 (7)	−0.0112 (7)	−0.0058 (6)	−0.0006 (6)
C3	0.0222 (10)	0.0207 (8)	0.0236 (8)	−0.0097 (7)	−0.0036 (7)	−0.0033 (6)
C4	0.0175 (9)	0.0177 (7)	0.0236 (7)	−0.0082 (7)	−0.0039 (6)	0.0023 (6)
C5	0.0125 (8)	0.0195 (7)	0.0173 (7)	−0.0070 (6)	−0.0005 (6)	0.0002 (5)
C6	0.0145 (9)	0.0184 (7)	0.0190 (7)	−0.0079 (6)	−0.0024 (6)	0.0005 (5)
C7	0.0196 (9)	0.0174 (7)	0.0198 (7)	−0.0090 (7)	−0.0068 (6)	0.0023 (5)
C8	0.0180 (9)	0.0189 (7)	0.0209 (7)	−0.0082 (7)	−0.0059 (6)	−0.0010 (5)
C9	0.0186 (9)	0.0222 (7)	0.0168 (6)	−0.0088 (7)	−0.0055 (6)	0.0013 (5)
C10	0.0185 (9)	0.0187 (7)	0.0203 (7)	−0.0077 (7)	−0.0065 (6)	0.0039 (6)
C11	0.0242 (10)	0.0168 (7)	0.0249 (8)	−0.0094 (7)	−0.0096 (7)	0.0021 (6)
C12	0.0190 (9)	0.0185 (7)	0.0327 (8)	−0.0041 (7)	−0.0099 (7)	−0.0018 (6)
C13	0.0183 (10)	0.0225 (7)	0.0271 (8)	−0.0068 (7)	−0.0033 (7)	−0.0062 (6)
C14	0.0233 (10)	0.0224 (7)	0.0192 (7)	−0.0094 (7)	−0.0032 (6)	−0.0030 (6)
C15	0.0238 (9)	0.0204 (7)	0.0223 (7)	−0.0136 (7)	−0.0073 (6)	0.0027 (6)
C16	0.0192 (9)	0.0225 (7)	0.0220 (7)	−0.0109 (7)	−0.0039 (6)	−0.0008 (6)
C17	0.0616 (17)	0.0366 (10)	0.0284 (9)	−0.0220 (11)	−0.0207 (9)	−0.0039 (8)
C18	0.0260 (12)	0.0494 (12)	0.0301 (9)	−0.0144 (10)	0.0006 (8)	−0.0009 (8)
C19	0.0212 (11)	0.0330 (9)	0.0361 (10)	−0.0103 (8)	−0.0114 (8)	0.0063 (8)
C20	0.0265 (10)	0.0319 (9)	0.0279 (8)	−0.0165 (8)	−0.0151 (7)	0.0089 (6)
C21	0.0248 (10)	0.0289 (8)	0.0231 (7)	−0.0154 (7)	−0.0135 (6)	0.0061 (6)
C22	0.0345 (11)	0.0277 (8)	0.0221 (8)	−0.0173 (8)	−0.0092 (7)	−0.0010 (6)
C23	0.0539 (14)	0.0422 (10)	0.0189 (7)	−0.0343 (10)	−0.0126 (8)	0.0060 (7)
C24	0.0414 (13)	0.0320 (9)	0.0415 (10)	−0.0220 (9)	−0.0240 (9)	0.0172 (8)
C25	0.0266 (12)	0.0313 (9)	0.0521 (12)	−0.0054 (8)	−0.0115 (9)	0.0063 (8)
C26	0.0218 (11)	0.0393 (10)	0.0324 (9)	−0.0106 (8)	−0.0032 (7)	0.0069 (7)

### Geometric parameters (Å, °)

**Table:**

N1—C19	1.457 (2)	C11—H11B	0.9900
N1—C16	1.4717 (19)	C12—C13	1.504 (2)
N1—C9	1.479 (2)	C12—H12	1.0000
O1—C1	1.3874 (18)	C13—C14	1.322 (3)
O1—C20	1.452 (2)	C14—H14	0.9500
O2—C2	1.3728 (19)	C15—C16	1.523 (2)
O2—C17	1.431 (2)	C15—H15A	0.9900
O3—C12	1.426 (2)	C15—H15B	0.9900
O3—H3	0.81 (3)	C16—H16A	0.9900
O4—C13	1.377 (2)	C16—H16B	0.9900
O4—C18	1.424 (2)	C17—H17A	0.98 (3)
C1—C6	1.405 (2)	C17—H17B	1.04 (2)
C1—C2	1.407 (2)	C17—H17C	0.94 (3)
C2—C3	1.384 (2)	C18—H18A	0.93 (3)
C3—C4	1.383 (2)	C18—H18B	0.97 (3)
C3—H3A	0.9500	C18—H18C	1.02 (3)
C4—C5	1.390 (2)	C19—H19A	1.00 (2)
C4—H4	0.9500	C19—H19B	0.95 (3)
C5—C6	1.408 (2)	C19—H19C	0.96 (2)
C5—C10	1.511 (2)	C20—C21	1.502 (2)
C6—C7	1.5414 (19)	C20—H20A	0.9900
C7—C11	1.537 (2)	C20—H20B	0.9900
C7—C8	1.5437 (19)	C21—C22	1.386 (2)
C7—C15	1.548 (2)	C21—C26	1.394 (3)
C8—C14	1.505 (2)	C22—C23	1.389 (2)
C8—C9	1.519 (2)	C22—H22	0.9500
C8—H8	1.0000	C23—C24	1.391 (3)
C9—C10	1.534 (2)	C23—H23	0.9500
C9—H9	1.0000	C24—C25	1.384 (3)
C10—H10A	0.9900	C24—H24	0.9500
C10—H10B	0.9900	C25—C26	1.382 (3)
C11—C12	1.531 (2)	C25—H25	0.9500
C11—H11A	0.9900	C26—H26	0.9500
			
C19—N1—C16	110.48 (13)	C14—C13—O4	126.33 (15)
C19—N1—C9	112.48 (12)	C14—C13—C12	123.58 (15)
C16—N1—C9	113.97 (12)	O4—C13—C12	110.06 (15)
C1—O1—C20	115.41 (11)	C13—C14—C8	122.38 (14)
C2—O2—C17	116.24 (13)	C13—C14—H14	118.8
C12—O3—H3	113.4 (19)	C8—C14—H14	118.8
C13—O4—C18	115.97 (15)	C16—C15—C7	110.79 (12)
O1—C1—C6	119.65 (12)	C16—C15—H15A	109.5
O1—C1—C2	118.96 (12)	C7—C15—H15A	109.5
C6—C1—C2	120.95 (13)	C16—C15—H15B	109.5
O2—C2—C3	123.84 (14)	C7—C15—H15B	109.5
O2—C2—C1	116.36 (13)	H15A—C15—H15B	108.1
C3—C2—C1	119.75 (13)	N1—C16—C15	111.43 (12)
C4—C3—C2	119.21 (13)	N1—C16—H16A	109.3
C4—C3—H3A	120.4	C15—C16—H16A	109.3
C2—C3—H3A	120.4	N1—C16—H16B	109.3
C3—C4—C5	121.95 (13)	C15—C16—H16B	109.3
C3—C4—H4	119.0	H16A—C16—H16B	108.0
C5—C4—H4	119.0	O2—C17—H17A	111.8 (14)
C4—C5—C6	119.53 (13)	O2—C17—H17B	110.5 (12)
C4—C5—C10	118.27 (13)	H17A—C17—H17B	110.3 (19)
C6—C5—C10	122.12 (12)	O2—C17—H17C	106.3 (14)
C1—C6—C5	118.22 (13)	H17A—C17—H17C	107 (2)
C1—C6—C7	121.07 (12)	H17B—C17—H17C	110.8 (19)
C5—C6—C7	119.75 (13)	O4—C18—H18A	105.7 (16)
C11—C7—C6	115.92 (13)	O4—C18—H18B	110.4 (13)
C11—C7—C8	105.00 (12)	H18A—C18—H18B	105 (2)
C6—C7—C8	112.20 (11)	O4—C18—H18C	111.8 (13)
C11—C7—C15	112.20 (12)	H18A—C18—H18C	112 (2)
C6—C7—C15	105.79 (12)	H18B—C18—H18C	112 (2)
C8—C7—C15	105.35 (12)	N1—C19—H19A	112.3 (14)
C14—C8—C9	112.17 (12)	N1—C19—H19B	107.5 (14)
C14—C8—C7	113.37 (12)	H19A—C19—H19B	104.5 (19)
C9—C8—C7	110.21 (12)	N1—C19—H19C	111.7 (14)
C14—C8—H8	106.9	H19A—C19—H19C	107.0 (18)
C9—C8—H8	106.9	H19B—C19—H19C	113.6 (19)
C7—C8—H8	106.9	O1—C20—C21	108.89 (12)
N1—C9—C8	108.56 (11)	O1—C20—H20A	109.9
N1—C9—C10	117.45 (12)	C21—C20—H20A	109.9
C8—C9—C10	107.64 (13)	O1—C20—H20B	109.9
N1—C9—H9	107.6	C21—C20—H20B	109.9
C8—C9—H9	107.6	H20A—C20—H20B	108.3
C10—C9—H9	107.6	C22—C21—C26	118.78 (15)
C5—C10—C9	114.41 (12)	C22—C21—C20	121.23 (16)
C5—C10—H10A	108.7	C26—C21—C20	119.93 (16)
C9—C10—H10A	108.7	C21—C22—C23	120.44 (17)
C5—C10—H10B	108.7	C21—C22—H22	119.8
C9—C10—H10B	108.7	C23—C22—H22	119.8
H10A—C10—H10B	107.6	C22—C23—C24	120.23 (16)
C12—C11—C7	114.76 (12)	C22—C23—H23	119.9
C12—C11—H11A	108.6	C24—C23—H23	119.9
C7—C11—H11A	108.6	C25—C24—C23	119.55 (16)
C12—C11—H11B	108.6	C25—C24—H24	120.2
C7—C11—H11B	108.6	C23—C24—H24	120.2
H11A—C11—H11B	107.6	C26—C25—C24	119.99 (19)
O3—C12—C13	112.56 (13)	C26—C25—H25	120.0
O3—C12—C11	109.93 (13)	C24—C25—H25	120.0
C13—C12—C11	111.95 (14)	C25—C26—C21	120.97 (17)
O3—C12—H12	107.4	C25—C26—H26	119.5
C13—C12—H12	107.4	C21—C26—H26	119.5
C11—C12—H12	107.4		
			
C20—O1—C1—C6	120.66 (16)	C14—C8—C9—C10	−61.20 (15)
C20—O1—C1—C2	−66.93 (18)	C7—C8—C9—C10	66.14 (15)
C17—O2—C2—C3	6.7 (3)	C4—C5—C10—C9	−159.57 (14)
C17—O2—C2—C1	−170.72 (17)	C6—C5—C10—C9	17.2 (2)
O1—C1—C2—O2	2.7 (2)	N1—C9—C10—C5	74.44 (17)
C6—C1—C2—O2	175.01 (16)	C8—C9—C10—C5	−48.35 (16)
O1—C1—C2—C3	−174.81 (16)	C6—C7—C11—C12	−63.95 (17)
C6—C1—C2—C3	−2.5 (2)	C8—C7—C11—C12	60.46 (16)
O2—C2—C3—C4	−171.90 (16)	C15—C7—C11—C12	174.37 (12)
C1—C2—C3—C4	5.4 (2)	C7—C11—C12—O3	87.15 (16)
C2—C3—C4—C5	−2.6 (2)	C7—C11—C12—C13	−38.74 (17)
C3—C4—C5—C6	−3.2 (2)	C18—O4—C13—C14	−3.6 (2)
C3—C4—C5—C10	173.62 (15)	C18—O4—C13—C12	174.42 (14)
O1—C1—C6—C5	169.01 (14)	O3—C12—C13—C14	−118.18 (17)
C2—C1—C6—C5	−3.2 (2)	C11—C12—C13—C14	6.3 (2)
O1—C1—C6—C7	0.3 (2)	O3—C12—C13—O4	63.72 (17)
C2—C1—C6—C7	−171.99 (15)	C11—C12—C13—O4	−171.84 (12)
C4—C5—C6—C1	6.0 (2)	O4—C13—C14—C8	178.32 (14)
C10—C5—C6—C1	−170.68 (14)	C12—C13—C14—C8	0.5 (2)
C4—C5—C6—C7	174.93 (13)	C9—C8—C14—C13	149.84 (14)
C10—C5—C6—C7	−1.8 (2)	C7—C8—C14—C13	24.21 (19)
C1—C6—C7—C11	−52.12 (19)	C11—C7—C15—C16	−172.80 (12)
C5—C6—C7—C11	139.31 (14)	C6—C7—C15—C16	59.89 (15)
C1—C6—C7—C8	−172.72 (14)	C8—C7—C15—C16	−59.11 (14)
C5—C6—C7—C8	18.7 (2)	C19—N1—C16—C15	178.44 (13)
C1—C6—C7—C15	72.91 (18)	C9—N1—C16—C15	−53.76 (16)
C5—C6—C7—C15	−95.66 (15)	C7—C15—C16—N1	55.26 (16)
C11—C7—C8—C14	−51.48 (15)	C1—O1—C20—C21	−175.36 (14)
C6—C7—C8—C14	75.26 (16)	O1—C20—C21—C22	−69.19 (19)
C15—C7—C8—C14	−170.11 (12)	O1—C20—C21—C26	113.59 (17)
C11—C7—C8—C9	−178.15 (12)	C26—C21—C22—C23	1.7 (3)
C6—C7—C8—C9	−51.41 (17)	C20—C21—C22—C23	−175.59 (15)
C15—C7—C8—C9	63.22 (14)	C21—C22—C23—C24	−0.2 (3)
C19—N1—C9—C8	−176.79 (13)	C22—C23—C24—C25	−1.0 (3)
C16—N1—C9—C8	56.45 (15)	C23—C24—C25—C26	0.8 (3)
C19—N1—C9—C10	60.90 (18)	C24—C25—C26—C21	0.6 (3)
C16—N1—C9—C10	−65.86 (16)	C22—C21—C26—C25	−1.9 (3)
C14—C8—C9—N1	170.71 (11)	C20—C21—C26—C25	175.41 (18)
C7—C8—C9—N1	−61.96 (15)		

### Hydrogen-bond geometry (Å, °)

**Table:**

*D*—H···*A*	*D*—H	H···*A*	*D*···*A*	*D*—H···*A*
O3—H3···N1^i^	0.81 (3)	2.20 (3)	2.8966 (17)	145 (2)

Symmetry codes: (i) *x*+1, *y*, *z*.
